# Genetic screening confirms heterozygous mutations in *ACAN* as a major cause of idiopathic short stature

**DOI:** 10.1038/s41598-017-12465-6

**Published:** 2017-09-22

**Authors:** Nadine N. Hauer, Heinrich Sticht, Sangamitra Boppudi, Christian Büttner, Cornelia Kraus, Udo Trautmann, Martin Zenker, Christiane Zweier, Antje Wiesener, Rami Abou Jamra, Dagmar Wieczorek, Jaqueline Kelkel, Anna-Maria Jung, Steffen Uebe, Arif. B Ekici, Tilman Rohrer, André Reis, Helmuth-Günther Dörr, Christian T. Thiel

**Affiliations:** 1 Institute of Human Genetics, Friedrich-Alexander-Universität Erlangen-Nürnberg (FAU), Erlangen, Germany; 2 Institute of Biochemistry, Friedrich-Alexander-Universität Erlangen-Nürnberg (FAU), Erlangen, Germany; 3 Institute of Human Genetics, Otto-von-Guericke University Magdeburg, Magdeburg, Germany; 4 Institute of Human Genetics, University of Leipzig, Leipzig, Germany; 5 Institute of Human Genetics, University of Duisburg-Essen, Essen, Germany; 6 Institute of Human-Genetics, Medical Faculty, Heinrich-Heine-University Duesseldorf, Duesseldorf, Germany; 7 Division of Pediatric Endocrinology, Department of Pediatrics and Neonatology, Saarland University Hospital, Homburg/Saar, Germany; 8 Department of Pediatrics and Adolescent Medicine, Friedrich-Alexander-Universität Erlangen-Nürnberg (FAU), Erlangen, Germany

## Abstract

Short stature is a common pediatric disorder affecting 3% of the population. However, the clinical variability and genetic heterogeneity prevents the identification of the underlying cause in about 80% of the patients. Recently, heterozygous mutations in the *ACAN* gene coding for the proteoglycan aggrecan, a main component of the cartilage matrix, were associated with idiopathic short stature. To ascertain the prevalence of *ACAN* mutations and broaden the phenotypic spectrum in patients with idiopathic short stature we performed sequence analyses in 428 families. We identified heterozygous nonsense mutations in four and potentially disease-causing missense variants in two families (1.4%). These patients presented with a mean of −3.2 SDS and some suggestive clinical characteristics. The results suggest heterozygous mutations in *ACAN* as a common cause of isolated as well as inherited idiopathic short stature.

## Introduction

**Figure 1:**
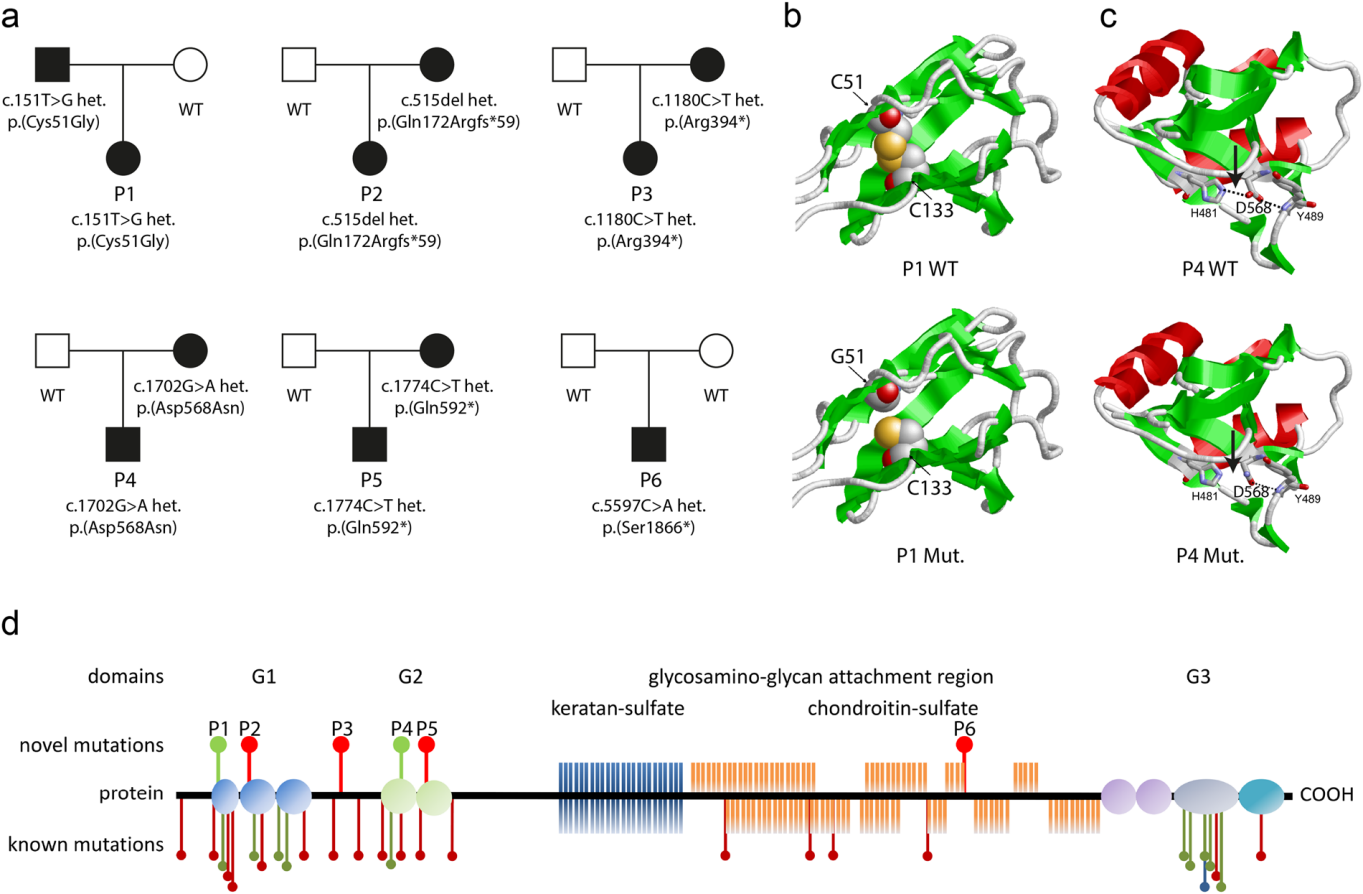
Mutations and protein structure of aggrecan. (**a**) Pedigrees of the six families with identified mutations in the *ACAN* gene. Both parents in families 2 und 3 presented with a height below/around −2 SDS. In both families the mother carrying the mutation was considered affected based on the phenotypic and radiographic evaluation (Table 1). (**b**) Model of the N-terminal Ig-domain of ACAN highlighting the site of the p.(Cys51Gly) mutation. In the wildtype (top model), cysteine at position 51 (C51) forms a disulfide bond with cysteine at position 133 (C133) that stabilizes the domain (cysteines are shown in space-filled presentation; the sulfur atoms of both cysteines that form the disulfide bond are shown in yellow). In the mutant variant (bottom model) this disulfide bond cannot be formed, which is expected to reduce domain stability of even cause unfolding of the entire domain. (**c**) Model of the third Link domain of ACAN highlighting the site of the p.(Asp568Asn) mutation. In the wildtype (top model), aspartate at position 568 (D568) forms two polar interactions (dotted lines) with the histidine at position 481 (H481) and the tyrosine at position 489 (Y489). In the mutant variant (bottom model) only one of these interactions can be formed, which is expected to reduce domain stability. (**d**) Aggrecan is a proteoglycan consisting of different functional domains (modified from Gkourogianni *et al*., 2016). The G1 domain (blue) consists of an immunglobulin-like repeat (oval) and two proteoglycan tandem repeats (orbital)^12^. The inter-globular domain separates it from the G2 domain (green) which consists of two proteoglycan tandem repeats (orbital). It is adjacent to the glycosaminoglycan attachment region (GAG) which carries keratan sulfate (dark blue) and chondroitin sulfate chains (orange). The C-terminal domain is the globular G3-domain which consists of two EGF-repeats (violet), a C-type lectin domain (grey) and a complement regulatory protein repeat (turquoise). In the upper part the localization of the identified novel heterozygous mutations (P1-6) is shown (green: missense mutation, red: nonsense mutation). In the lower part, all previously reported mutations are shown (see Supplementary Table 1) (green: heterozygous missense mutation, red: heterozygous nonsense mutation, blue: homozygous missense mutation).

Short stature is defined as a height of at least two standard deviations below the population specific age- and sex-related average^1^. It might occur either with regular body proportions or disproportionate, the latter observed in most forms of skeletal dysplasias affecting the growth of distinct bones^1,2^. The longitudinal growth of the bones is mainly regulated by the configuration of the growth plate^3^. The growth plate is embedded between epi- and metaphysis and is composed of the resting, proliferative and hypertrophic zone. The resting zone consists of the chondrocyte progenitor cells^3–5^. These undergo cell division in the proliferative zone, differentiate to chondrocytes and terminate proliferation in the hypertrophic zone. Osteoblasts, osteoclasts and blood vessels transform the newly formed cartilage into bone. Aggrecan is the main proteoglycan of the extracellular matrix of the growth plate cartilage^6^. Mutations in *ACAN*, which encodes for aggrecan, are associated with growth defects ranging from mild idiopathic short stature to severe skeletal dysplasias [MIM: 165800, 612813, 608361] (Fig. 1d)^7^. Biallelic mutations lead to spondyloepimetaphyseal dysplasia comprising severe short stature with a final adult height between 66 and 71 cm, brachydactyly and characteristic clinical findings [MIM: 612813]^8^. Parents carrying heterozygous mutations present with a final height of 150–152 cm without further dysmorphic findings^8^. Although different in their entity, all described phenotypes caused by mutations in the *ACAN* gene include reduced height of the patients. This phenotypic spectrum suggests a dosage effect. Recently, the phenotypic spectrum arising from heterozygous mutations in *ACAN* was evaluated in 103 patients from 20 selected families^9^. Currently, no systematic data is present about the frequency of heterozygous *ACAN* mutations in patients with idiopathic short stature in a larger cohort of mostly European decent.

In this report, we systematically analyzed 428 families to establish the prevalence of heterozygous mutations in *ACAN* in idiopathic short stature.

## Results and Discussion

Short stature is a heterogeneous trait^10^. The most common underlying monogenic cause are defects, deletions and mutations, of the SHOX gene attributing for 2.4% of patients with idiopathic short stature^11^.

To identify the proportion of heterozygous defects in *ACAN* we analyzed 428 patients with idiopathic short stature and identified potential disease causing mutations in 6 patients (1.4%) (Fig. 1, Table 1). Four of them were truncating variants including one frameshift variant leading to a premature termination codon. The missense variant p.(Cys51Gly) in patient P1 is located at an evolutionary highly conserved position and has a CADD score of 18.27 suggesting a deleterious effect on the protein. This missense variant affects the region coding for the globular domain G1 exhibiting an Immunoglobulin(Ig) fold. The analysis of the protein structure of the missense variant p.(Cys51Gly) revealed that Cys51 forms a disulfide bond with Cys133 (Fig. 1b). This interaction represents the canonical disulfide bond, which is present in most Immunoglobulin folds and plays an important role for domain stability. A replacement of Cys51 by glycine leads to a loss of this disulfide bond (Fig. 1b) and was therefore expected to cause a significant reduction of domain stability. As the G1 domain has been reported to be functionally essential for the localization of the proteoglycan to the specific tissue by linking aggrecan to hyaluronan we expect that the missense variant might interfere with this function suggesting a loss of function effect^12^. The missense variant p.(Asp568Asn) in patient P4 is located in the third link domain (residues 478–573), a hyaluronan-binding protein module. The variant affects the C-terminus of the domain where its sidechain forms two polar interactions with the Tyr489 backbone and the His481 sidechain located in the N-terminal region of the domain (Fig. 1c). In the p.(Asp568Asn) variant, the sidechain group is altered from a carboxyl group to an amide group, which can only form one of the two polar interactions observed in the wildtype. Thus, the interaction between the C-terminus and the N-terminus is weakened, which is expected to destabilize the entire domain thereby also affecting its ligand binding properties. All variants were therefore characterized as likely pathogenic or pathogenic based on the ACMG criteria^13^.

**Table 1:** Phenotype of patients with *ACAN* mutations.

	P1	P2	P3	P4	P5	P6
*ACAN* Mutation	c.151 T>G p.(Cys51Gly)	c.515del p.(Gln172Argfs*59)	c.1180 C>T p.(Arg394*)	c.1702 G>A p.(Asp568Asn)	c.1774 C>T p.(Gln592*)	c.5597 C>A p.(Ser1866*)
Inheritance	paternal	maternal	maternal	maternal	maternal	de novo
Gender	female	female	female	male	male	male
SGA	X					
Height (SDS)	−3.5	−3.6	−3.9	−3.2	−3.2	−2.0
Height mother/ father (SDS)	−1.1/−3.2	−3.8/−2.4	−1.9/−2.3	−2.0/−1.8	−1.8/−0.7	−0.3/−1.2
Stature type	proportionate	proportionate	proportionate	proportionate	proportionate	proportionate
Bone age	accelerated	delayed	delayed	delayed	na	accelerated
Osteochondritis dissecans			X (maternal)	X	na	
Head circumference (SDS)	0.5	−1.6	−1.7	−0.2	1.6	2.7
Arm span height ratio	1	1	1	1	na	1
Prominent forehead		X	X			X
Short neck		X	X		X	X
Barrel-shaped chest	X	X	X	X	X	X
Limited supination	X	X	X	X		
Brachydactyly		X	X			X

Four of the variants were maternally inherited, one paternally inherited and one variant occurred *de novo* (Fig. 1a). In family P2 the mother presented with a height of −3.8 SDS and the father with −2.4 SDS. Here, the mutation co-segregated with the phenotypic and radiographic characteristics in the mother. In family P3 the nonsense variant p.(Arg394*) in *ACAN* was inherited from the mother with a height of −1.9 SDS. Both the patient P3 and the mother, as well as the maternal grandmother showed radiographic signs of osteochondritis dissecans and the phenotype was therefore considered to be maternally inherited. We further identified a potential pathogenic variant in the natriuretic peptide receptor 2 (NPR2, NM_003995.3:c.941 T>A; p.(Leu314Gln)) in this patient. This variant has not been reported in ExAC, affects a highly conserved amino acid, and was inherited from the father with a height of −2.3 SDS. As heterozygous mutations in *NPR2* were also shown to cause short stature without specific skeletal abnormalities^14^, we propose a combined negative effect of both variants on the growth phenotype of patient P3. The observation of mutations in more than one gene contributing to a patient’s phenotype has recently been reported for other diseases^15^.

**Figure 2:**
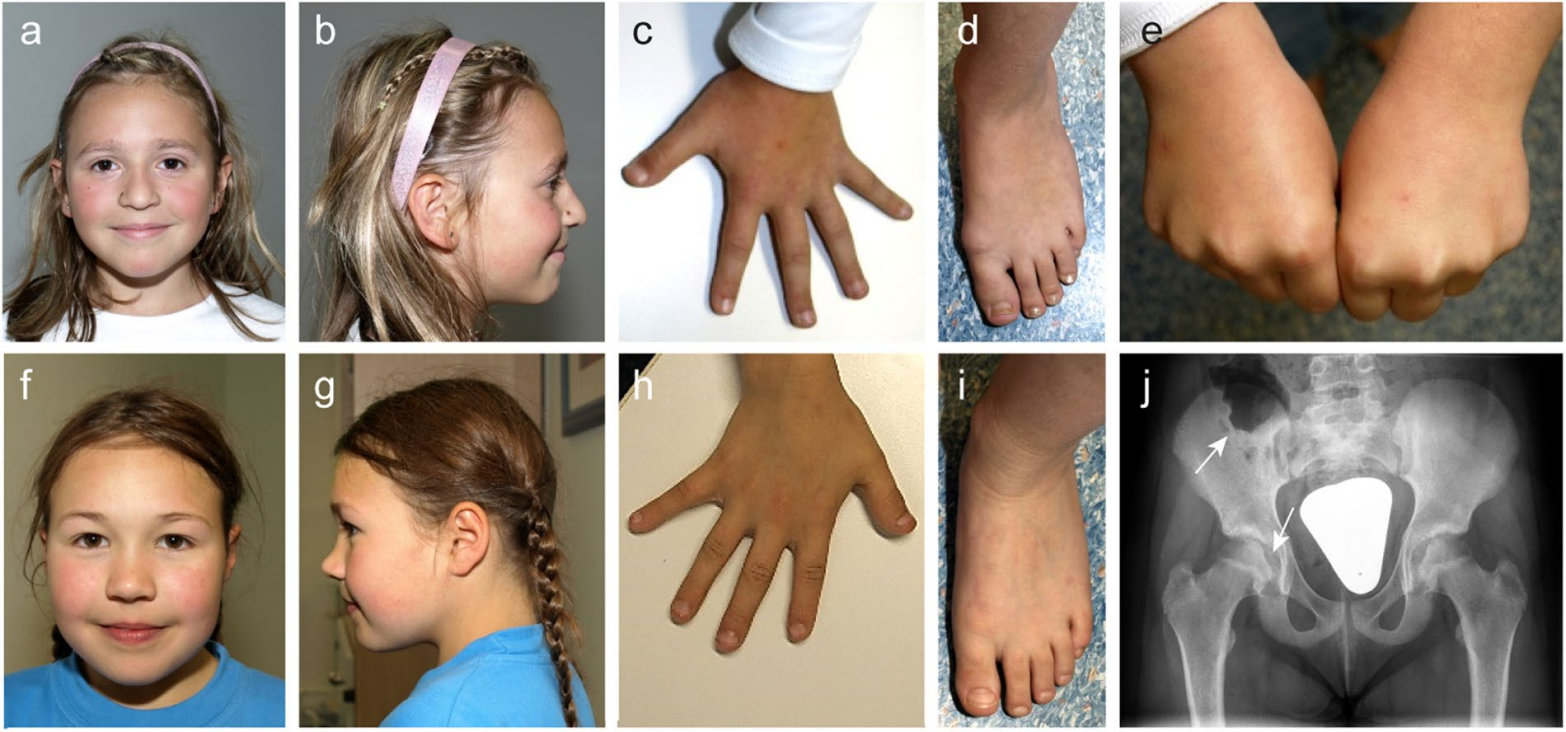
Clinical and radiographic characteristics of two patients with heterozygous ACAN mutations. (**a**–**e**) Patient P2 was born at term with a weight of 3270 g and a body length of 48 cm. At the age of 10 years she had a height of 119.6 cm (−3.6 SDS), weight of 25.2 kg (BMI 17) and head circumference of 50 cm (−1.6 SDS) (**f**–**j**) Patient P3 was born at term with a weight of 3550 g and a body length of 50 cm. At the age of 11 years she presented with a height of 121 cm (−3.9 SDS), weight of 28.4 kg (BMI 19) and head circumference of 50 cm (−1.7 SDS). Psychomotor development was normal in both patients. Both presented with proportionate short stature, broad chest, short neck, brachydactyly with pronounced brachymetacarpaly V, broad thumb and great toe and an inhibition of extension of the elbow. (**a**,**b**,**f**,**g**) Facial anomalies include a broad nasal tip and a high forehead. (**j**) Radiographic evaluation revealed signs of Osteochondritis dissecans (arrows) in patient P3, her mother and maternal grandmother.

The herewith reported six patients all presented with proportionate short stature between 2 and 4 SDS below the average accompanied by brachydactyly in patient 2, 3 and 6 (Fig. 2, Table 1). To date, 27 different heterozygous mutations in *ACAN* have been reported to cause different entities of short stature (Fig. 1d and Supplementary Table 2)^9,16–26^. Eight of these mutations were frameshift variants, seven were missense variants, eleven were nonsense variants and one was a splice site variant. The patients’ height varied between 0.9 and 5.9 SDS below the average, whereby no final relation between the mutation type and the extent of the clinical characteristics was identified. Most of these patients presented with an advanced bone age and proportionate to mildly disproportionate short stature, some had a primordial growth retardation and a distinct facial gestalt including midfacial hypoplasia and a flat nasal bridge, broad great toes, early-onset osteoarthritis, intervertebral disc disease or brachydactyly. Their skeletal phenotypes varied between absent and mild skeletal dysplasia including Osteochondritis dissecans. Three of the patients in this study (P2, P3 and P4) showed a delayed bone age (Table 1). This suggests that the bone age of an individual has no predictive value in patients with ACAN mutations. Whereas a recent publication suggested primordial growth retardation as a positive predictive value for the identification of ACAN mutations in patients^17^, this holds true for only one of our patients (P1). A distinct facial gestalt as described before^9,17^ was present in patients 2 and 3, but not obviously in the other patients (Fig. 2). Common but yet unreported symptoms include a barrel-shaped chest (P1, P2, P3, P5 and P6) and a limited supination of the forearm (P1, P2 and P3).

Some evidence for the response to growth hormone treatment was reported just recently^9,17,18^. Of the patients in this study, only patient P4 received growth hormone therapy improving his height from −3,2 SDS to −1,7 SDS. This underlines the need for randomized trials proving this effect in further affected individuals.

In summary, we systematically identified heterozygous mutations in *ACAN* as the second most common monogenic cause of idiopathic short stature (1.4%) just following the prevalence of *SHOX* gene defects. This was recently confirmed by the identification of the same frequency of *ACAN* mutations in a smaller group of Chinese patients^19^. These results imply to consider *ACAN* mutations in the genetic evaluation of patients with idiopathic short stature even in the absence of characteristic features of a skeletal dysplasia.

## Methods

### Patient information

We analyzed the *ACAN* gene in a group of 428 families of mostly European descent selected because of short stature (for detailed information see Supplementary Appendix). The patients’ height ranged between −1.49 and −9.9 SDS (Median: −3.3 SDS) below the age-related average. 86% presented with proportionate short stature, 14% with disproportionate short stature. In 36%, we identified accompanying clinical signs (syndromic short stature), whereas 64% were individuals with isolated short stature. 28% of the patients had a prenatal onset of growth retardation. Regarding occipitofrontal circumference, 1% of the patients were macrocephalic, 75% normocephalic and 24% were microcephalic.

All families gave written informed consent for study participation and publication of identifying information/images. This study has been approved by the ethical committee of the Medical Faculty of the Friedrich-Alexander-Universität Erlangen-Nürnberg (No. 180_15 Bc) and the ethical committee of the medical association of Saarland (No. 58/06) and conducted in accordance with these guidelines and the Declaration of Helsinki principles.

### Genetic analyses

We isolated DNA from peripheral blood of patients and all available family members and performed whole exome sequencing for 200 of these patients and evaluated the exomes with regard to variants in *ACAN* (see Supplementary Appendix). In 120 patients the *ACAN* gene was analyzed as part of a multigene panel using an Illumina Nextera® Rapid Capture CustomKit. This multigene panel included a total of 329 genes related to short stature and RAS-MAPK signaling (a complete list of genes is available on request). In 108 families, we sequenced the *ACAN* gene by Sanger sequencing. As in other studies, a complex repetitive region within the coding region was excluded from the analysis (Supplementary Figure 1)^25^. The resulting variants were classified in accordance with the ACMG criteria^13,27,28^. Variants of possible pathogenic impact were confirmed by Sanger sequencing and their segregation in respective families was further evaluated.

Known genetic causes of growth retardation in the patients where we identified *ACAN* mutations were excluded by detailed clinical evaluation followed by targeted sequencing.

### Protein model

Templates for modelling the p.(Cys51Gly) variant of ACAN were identified using the profile-profile alignment tool HHpred^29^. For structural modeling the most similar template was used, which is the Immunoglobulin-domain of the hCAR receptor (PDB code: 1EAJ)^30^. The Link domain carrying the p.(Asp568Asn) variant was modeled based on the homologous Link_TSG6 domain (PDB code: 2PF5)^31^. Modelling was performed with Modeller9.16 and RasMol was used for structure analysis and visualization^32,33^.

### Data availability

The datasets generated during and/or analysed during the current study are available from the corresponding author on reasonable request.

## Electronic supplementary material

Supplementary information
