# Supplementary material for: Genetic screening confirms heterozygous mutations in ACAN as a major cause of idiopathic short stature
Source: Sci Rep. 2017 Sep 22;7:12225. doi: 10.1038/s41598-017-12465-6 (PMC5610314; doi:10.1038/s41598-017-12465-6)
Supplement: Supplementary file 1 — Supplementary information [file 41598_2017_12465_MOESM1_ESM.pdf]

Supplementary Material and Methods

Genetic screening confirms heterozygous mutations in *ACAN* as a major cause of idiopathic short stature

Nadine N Hauer[1], Heinrich Sticht[2], Sangamitra Boppudi[3], Christian Büttner[1],Cornelia Kraus[1], Udo Trautmann[1], Martin Zenker[3], Christiane Zweier[1], Antje Wiesener[1], Rami Abou Jamra[4], Dagmar Wieczorek[5,6], Jaqueline Kelkel[7], Anna-Maria Jung[7] Steffen Uebe[1], Arif B Ekici[1], Tilman Rohrer[7], André Reis[1], Helmuth-Günther Dörr[8], Christian T Thiel[1]

[1]Institute of Human Genetics, Friedrich-Alexander-Universität Erlangen-Nürnberg (FAU), Erlangen, Germany
[2]Institute of Biochemistry, Friedrich-Alexander-Universität Erlangen-Nürnberg (FAU), Erlangen, Germany
[3]Institute of Human Genetics, Otto-von-Guericke University Magdeburg, Magdeburg, Germany
[4]Institute of Human Genetics, University of Leipzig, Leipzig, Germany
[5]Institute of Human Genetics, University of Duisburg-Essen, Essen, Germany
[6]Institute of Human-Genetics, Medical Faculty, Heinrich-Heine-University Duesseldorf, Duesseldorf, Germany
[7]Division of Pediatric Endocrinology, Department of Pediatrics and Neonatology, Saarland University Hospital, Homburg/Saar, Germany
[8]Department of Pediatrics and Adolescent Medicine, Friedrich-Alexander-Universität Erlangen-Nürnberg (FAU), Erlangen, Germany

Supplementary methods:

Patients

In this study, we included patients with idiopathic short stature defined by a height of 2 SD below the population specific age- and sex-related average or 2 SD below the estimated target family height. All patients have been evaluated by experts in pediatric endocrinology and medical genetics to exclude identifiable known causes of short stature including growth hormone deficiency.

Whole exome sequencing & variant evaluation

Whole exome sequencing was performed for 200 of the patients after enrichment accomplished by SureSelect targeted capturing. Sequencing was conducted on a SOLiD or HiSeq2500 device and image analysis as well as base calling was performed using the corresponding software with default parameters. We performed read alignment with BWA[1] version 0.7.8 to the human genome assembly hg19 (GRCh37). Local re-alignment around potential InDel sites was performed with the Genome Analysis Toolkit[2] version 3.1. Single-nucleotide variants and small insertions and deletions (indels) were detected using five different callers: HaplotypeCaller and UnifiedGenotyper[2], SNVer[3], freeBayes[4] and Platypus[5]. Variant annotation was performed using ANNOVAR.[6] We achieved an average SureSelect target coverage of 160x and average coverage of the *ACAN* gene of 137.4x (Supplementary Figure 1). We included only variants called with GATKHap, GATKUG[2,7] or SNVer[3] which were covered by at least 10 % of the average coverage of the patient's exome and for which at least 5 novel alleles were detected. We then confirmed the selected variants (Combined Annotation Dependent Depletion (CADD) score >10, frequency in Exome Aggregation Consortium (ExAC) database $<10^{-3}$)[8,9] and their inheritance by Sanger Sequencing. 120 patients were analyzed by multigene panel sequencing using an Illumina Nextera® Rapid Capture CustomKit for enrichment and an Illumina MiSeq system for analysis. This multigene panel included a total of 329 genes related to short stature and RAS-MAPK signaling (a complete list of genes is available on request). The obtained sequence data covered all exons and the adjacent intronic nucleotides of *ACAN* with a minimal coverage of >40x. Variants were filtered according to population frequency and predicted impact on the gene product using the Illumina's VariantStudio v2.2 and Alamut software suite (Interactive Biosoftware, Rouen, France) software tools. Rare or novel variants of possible pathogenic impact were confirmed by Sanger sequencing and their segregation in respective families was further evaluated.

Supplementary Table 1: ACMG Scoring of identified disease-related variants in *ACAN*

**Patient**	**cDNA level (NM_013227.3)**	**Protein level**	**ACMG Subcategories**	**ACMG prediction**
P1	c.151T>G	p.(Cys51Gly)	PM2, PM7, PP1, PP2, PP3, PP4	Likely pathogenic (V)
P2	c.515del	p.(Gln172Argfs*59)	PVS1, PM2, PM7, PP1, PP4	Pathogenic (Ib)
P3	c.1180C>T	p.(Arg394*)	PVS1, PM2, PM7, PP1	Pathogenic (Ib)
P4	c.1702G>A	p.(Asp568Asn)	PM7, PP1, PP2, PP3, PP4	Likely pathogenic (VI)
P5	c.1774C>T	p.(Gln592*)	PVS1, PM2, PM7, PP1, PP4	Pathogenic (Ib)
P6	c.5597C>A	p.(Ser1866*)	PVS1, PS2, PM2, PM7, PP4	Pathogenic (Ia)

Supplementary Table 2: Overview of *ACAN mutations* (see also Figure 1c)

cDNA level (NM_013227.3)*	Protein level*	ACMG Category	Affected domain	Inheritance	Reference
c.6_13del	p.(Thr3Leufs*21)	na	na	paternal	[10]
c.61G>T	p.(Glu21*)	na	G1	maternal	[11]
c.151T>G	p.(Cys51Gly)	Likely pathogenic	G1	paternal	P1
c.223T>C	p.(Trp75Arg)	na	G1	maternal	[11]
c.272delA	p.(Arg93Alafs*41)	na	G1	maternal	[11,12]
c.492C>G	p.(Tyr164*)	na	G1	maternal	[11]
c.515del	p.(Gln172Argfs*59)	Pathogenic	G1	maternal	P2
c.532A>T	p.(Asn178Tyr)	na	G1	maternal	[11]
c.661del	p.(Tyr221Metfs*10)	na	G1	maternal	[10]
c.903G>C	p.(Trp301Cys)	na	G1	paternal	[11]
c.916A>T	p.(Ser306Cys)	na	G1	maternal	[11]
c.1047_1048delinsAC	p.(Tyr349*)	na	G1	maternal	[11]
c.1120_1123del	p.(Thr374*)	na	IGD	paternal	[10]
c.1180C>T	p.(Arg394*)	Pathogenic	IGD	maternal	P3
c.1443G>T	p.(Glu415*)	na	IGD	maternal	[11]
c.1425delA	p.(Val478Serfs*14)	na	G2	paternal	[11]
c.1526C>A	p.(Ser509*)	na	G2	paternal	[11]
c.1608C>A	p.(Tyr536*)	na	G2	maternal	[13]
c.1702G>A	p.(Asp568Asn)	Likely pathogenic	G2	maternal	P4
c.1744delT	p.(Phe582Serfs*69)	na	G2	maternal	[14]
c.1774C>T	p.(Gln592*)	Pathogenic	G2	maternal	P5
c.2026+1G>A	p.?	na	G2	maternal	[11,12]
c.3758dupC	p.(Gly1254Trpfs*175)	na	GAG	unknown	[15-17]
c.4657G>T	p.(Glu1553*)	na	GAG	maternal	[11]
c.4762_4765del	p.(Gly1588Cysfs*26)	na	GAG	paternal	[13]
c.5391delG	p.(Gln1798Serfs*53)	na	GAG	maternal	[11,18]
c.5597C>A	p.(Ser1866*)	Pathogenic	GAG	de novo	P6

c.7064T>C	p.(Leu2355Pro)	na	G3	maternal	[11,12]
c.7090C>T	p.(Gln2364*)	na	G3	maternal	[13]
c.7141G>A	p.(Asp2381Asn)	na	G3	maternal & paternal	[19]
c.7153G>A	p.(Glu2385Lys)	na	G3	maternal	[11]
c.7203G>A	p.(Trp2401*)	na	G3	paternal	[11]
c.7249G>A	p.(Val2417Met)	na	G3	maternal	[11,20,21]
c.7276G>T	p.(Glu2426*)	na	G3	maternal	[11]

* All variants have been updated to the actual recommendations of the "Human Genome Variation Society"[22] using the Mutalyzer software[23].

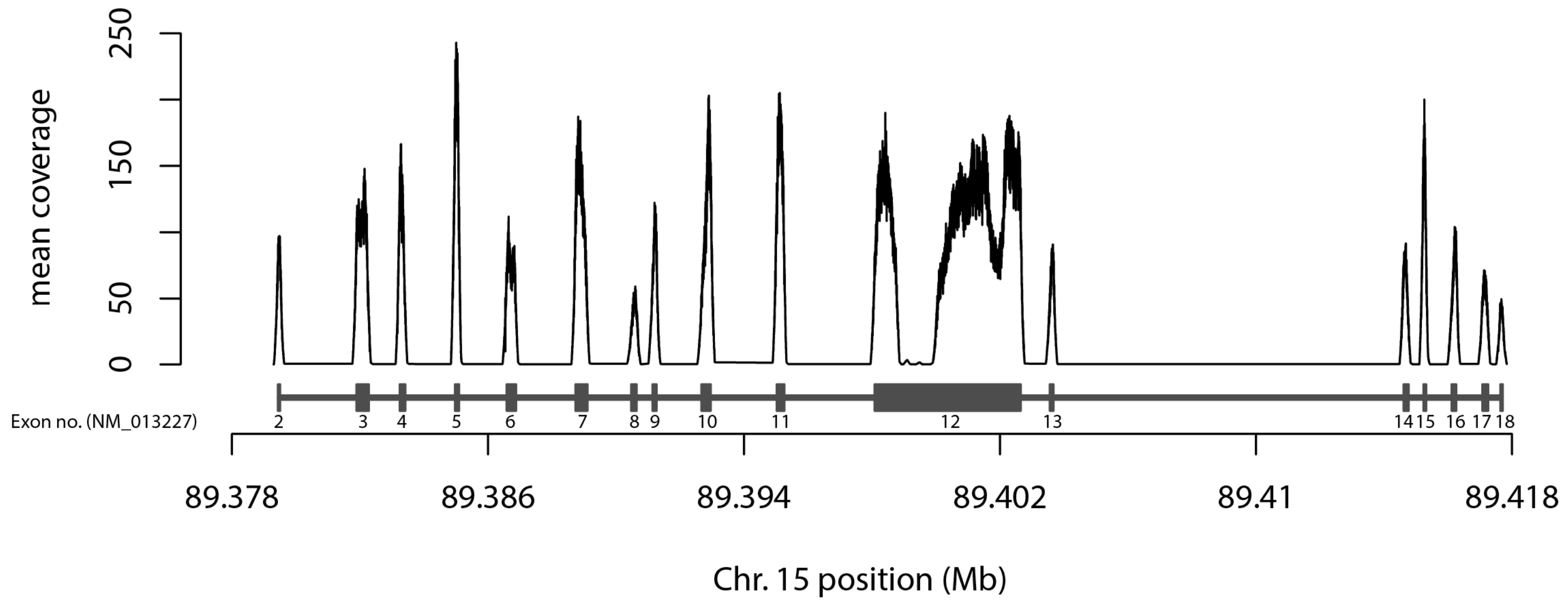

Supplementary Figure 1: Mean coverage of the coding regions of the ACAN gene from whole exome analysis. All coding exons and the conserved splice-sites were included in the analysis (read depth at least 20x). A complex repetitive region within the coding region (exon 12) was excluded from the analysis.

Supplementary References

1. Li, H. & Durbin, R. Fast and accurate short read alignment with Burrows-Wheeler transform. *Bioinformatics* **25**, 1754-60 (2009).
2. McKenna, A. *et al.* The Genome Analysis Toolkit: a MapReduce framework for analyzing next-generation DNA sequencing data. *Genome Res* **20**, 1297-303 (2010).
3. Wei, Z., Wang, W., Hu, P., Lyon, G.J. & Hakonarson, H. SNVer: a statistical tool for variant calling in analysis of pooled or individual next-generation sequencing data. *Nucleic Acids Res* **39**, e132 (2011).
4. Garrison, E. & Marth, G. Haplotype-based variant detection from short-read sequencing. (2012).
5. Rimmer, A. *et al.* Integrating mapping-, assembly- and haplotype-based approaches for calling variants in clinical sequencing applications. *Nat Genet* **46**, 912-8 (2014).
6. Wang, K., Li, M. & Hakonarson, H. ANNOVAR: functional annotation of genetic variants from high-throughput sequencing data. *Nucleic Acids Res* **38**, e164 (2010).
7. DePristo, M.A. *et al.* A framework for variation discovery and genotyping using next-generation DNA sequencing data. *Nat Genet* **43**, 491-8 (2011).
8. Kircher, M. *et al.* A general framework for estimating the relative pathogenicity of human genetic variants. *Nat Genet* **46**, 310-5 (2014).
9. Lek, M. *et al.* Analysis of protein-coding genetic variation in 60,706 humans. *bioRxiv* (2016).
10. Hu, X. *et al.* Novel pathogenic ACAN variants in non-syndromic short stature patients. *Clin Chim Acta* **469**, 126-129 (2017).
11. Gkourogianni, A. *et al.* Clinical characterization of patients with autosomal dominant short stature due to aggrecan mutations. *J Clin Endocrinol Metab*, jc20163313 (2016).
12. Nilsson, O. *et al.* Short stature, accelerated bone maturation, and early growth cessation due to heterozygous aggrecan mutations. *J Clin Endocrinol Metab*, jc20141332 (2014).
13. van der Steen, M. *et al.* ACAN Gene Mutations in Short Children Born SGA and Response to Growth Hormone Treatment. *J Clin Endocrinol Metab* **102**, 1458-1467 (2017).
14. Dateki, S. *et al.* Identification of a novel heterozygous mutation of the Aggrecan gene in a family with idiopathic short stature and multiple intervertebral disc herniation. *J Hum Genet* **62**, 717-721 (2017).
15. Gleghorn, L., Ramesar, R., Beighton, P. & Wallis, G. A mutation in the variable repeat region of the aggrecan gene (AGC1) causes a form of spondyloepiphyseal dysplasia associated with severe, premature osteoarthritis. *Am J Hum Genet* **77**, 484-90 (2005).
16. Anderson, I.J. *et al.* Spondyloepiphyseal dysplasia, mild autosomal dominant type is not due to primary defects of type II collagen. *Am J Med Genet* **37**, 272-6 (1990).
17. Eyre, S. *et al.* Identification of a locus for a form of spondyloepiphyseal dysplasia on chromosome 15q26.1: exclusion of aggrecan as a candidate gene. *J Med Genet* **39**, 634-8 (2002).
18. Quintos, J.B., Guo, M.H. & Dauber, A. Idiopathic short stature due to novel heterozygous mutation of the aggrecan gene. *J Pediatr Endocrinol Metab* (2015).
19. Tompson, S.W. *et al.* A recessive skeletal dysplasia, SEMD aggrecan type, results from a missense mutation affecting the C-type lectin domain of aggrecan. *Am J Hum Genet* **84**, 72-9 (2009).
20. Stattin, E.L. *et al.* A missense mutation in the aggrecan C-type lectin domain disrupts extracellular matrix interactions and causes dominant familial osteochondritis dissecans. *Am J Hum Genet* **86**, 126-37 (2010).
21. Stattin, E.L., Tegner, Y., Domellof, M. & Dahl, N. Familial osteochondritis dissecans associated with early osteoarthritis and disproportionate short stature. *Osteoarthritis Cartilage* **16**, 890-6 (2008).
22. den Dunnen, J.T. & Antonarakis, S.E. Mutation nomenclature extensions and suggestions to describe complex mutations: a discussion. *Hum Mutat* **15**, 7-12 (2000).

23. Wildeman, M., van Ophuizen, E., den Dunnen, J.T. & Taschner, P.E. Improving sequence variant descriptions in mutation databases and literature using the Mutalyzer sequence variation nomenclature checker. *Hum Mutat* **29**, 6-13 (2008).
